# Combined use of spatial restraint stress and middle cerebral artery occlusion is a novel model of post-stroke depression in mice

**DOI:** 10.1038/srep16751

**Published:** 2015-11-17

**Authors:** Gaocai Zhang, Li Chen, Lingli Yang, Xiaodong Hua, Beiqun Zhou, Zhigang Miao, Jizhen Li, Hua Hu, Michael Namaka, Jiming Kong, Xingshun Xu

**Affiliations:** 1 Department of Neurology, the Second Affiliated Hospital of Soochow University, Suzhou City, China; 2 Institute of Neuroscience, Soochow University, Suzhou City, China; 3 Department of Biochemistry, Franklin College of Arts and Sciences, University of Georgia, Athens, GA, USA; 4 Department of Neurology, Suzhou Kowloon Hospital, 118 Wansheng Street, Suzhou City, China; 5 College of Pharmacy and Medicine, University of Manitoba, Winnipeg, Manitoba, Canada; 6 Department of Human Anatomy and Cell Science, University of Manitoba, Winnipeg, Manitoba, Canada

## Abstract

Post stroke depression (PSD) is one of the most common complications of ischemic stroke. At present, the underlying mechanisms are unclear, largely because there are no reliable, valid and reproducible animal models of PSD. Here we report a novel animal model of PSD that displays consistent and reliable clinical features of hemiplegic stroke. The animal model encompasses a combination of the middle cerebral artery occlusion (MCAO) and spatial restraint stress. We found that a 60-minute MCAO followed by spatial restraint stress for 2 h daily for 2 to 4 weeks from the fourth day after MCAO induced PSD-like depressive phenotypes in mice. Importantly, the mice showed exacerbated deficits of neurological functions and decreased body weights, which were accompanied with reduced levels of brain derived neurotrophic factor and neurotransmitters including serotonin and dopamine. In addition, we identified increased levels of serum cortisol in our PSD mice. Finally, we found that mice with PSD were responsive to the tri-cyclic antidepressant imipramine as evidenced by their attenuated depressive behaviors, increased body weights, recovered brain serotonin levels, and decreased serum cortisol levels. This mouse model replicates multiple features of human post-stroke depression and thus provides a new model for the investigation of PSD.

Ischemic stroke is a leading cause of death and disabilities that include sensory, motor, and cognitive deficits^1^. Post-stroke depression (PSD) is one of the most common complications of ischemic stroke. PSD is characterized by depression and other mood and/or behavioral changes^2^. In China, the incidence of PSD ranges from 23.0 to 76.1%^3^; however, the underlying mechanism of PSD is still unclear.

The middle cerebral artery occlusion (MCAO) animal model has been widely used to study ischemic brain injury^4^,^5^,^6^; however, most studies indicate that there are no spontaneous post-stroke depressive-like symptoms in the mice^7^,^8^,^9^. Current PSD models are largely based on the combination of MCAO and unpredictable chronic mild stress (CMS)^10^,^11^. Social isolation for several weeks before MCAO is also used to induce PSD^12^. However, since PSD often occurs from 2 months to 1 year after the onset of stroke^13^,^14^,^15^, these models are not truly reflective of the usual course of clinical events in post-stroke depression, thereby warranting the need to develop a new model that truly reflects that usual time course of events.

Patients that suffer hemiplegia following an ischemic stroke become highly dependent on caregivers as a result of their mobility deficits. As such, they experience secondary physical restraint and psychological stress, which may contribute to the development of depressive symptoms typically observed to occur 6–8 weeks after the initial stroke. Applying this theory, previous studies have demonstrated that spatial restraint stress is a good animal model for examining the structure and function of the hippocampus^16^,^17^. To mimic the clinical course of PSD, we established a novel animal model that encompasses the combination of MCAO model along with spatial restraint stress.

## Results

### Determination of an optimal ischemic duration of MCAO

**Figure 1:**
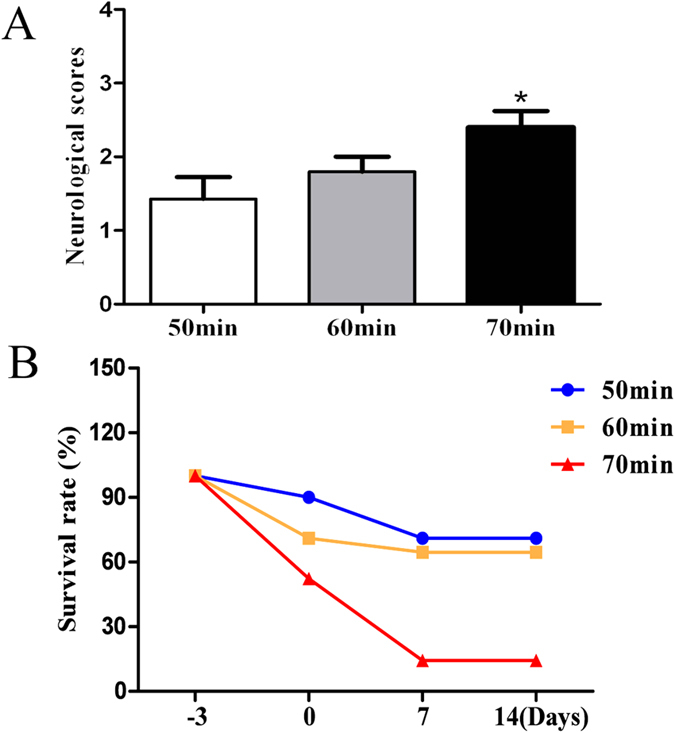
Different periods of MCAO were evaluated by neurological scores and mouse survival after stroke. Neurological scores were examined after different time points (50, 60, or 70 min) of MCAO and 24 h of reperfusion (**A**). The rate of mortality of mice was recorded for 2 weeks in mice with different ischemia times (**B**). *P < 0.05 versus 50 min group. N = 11.

To find an optimal ischemic duration of MCAO for a PSD model, we examined the neurological scores after different ischemic times (50, 60, or 70 min) and 24 h of reperfusion. The data showed that a longer ischemic time resulted in higher neurological scores (P < 0.05, Fig. 1A). Specifically, 70 min of ischemia caused more severe brain damage. Therefore, we further evaluated the survival rate within 2 weeks after MCAO. In the group with 70 min of ischemia, the survival rate was 15%, while the survival rates were 65% and 72% in 50 min and 60 min groups, respectively (Fig. 1B). Because of high mortality, 70 minutes of MCAO was considered not suitable for this PSD model. Because there was no statistically significant difference in survival rates between the 50 min and 60 min groups, both groups were further examined.

**Figure 2:**
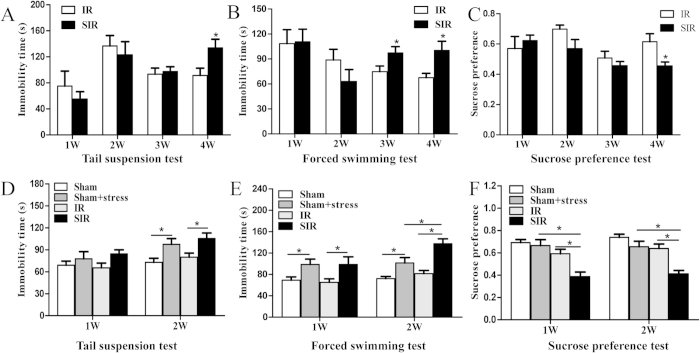
Depressive behaviors were evaluated after different MCAO ischemia time and spatial restraint stress. After mice were subjected to MCAO for 50 min or 60 min, different behavioral tests were examined weekly. Immobility time in tail suspension test and forced swimming test was recorded in mice with 50 min of MCAO (**A**,**B**) and the mice with 60 min of MCAO (**D**,**E**). The percentage of 1% sucrose consummation in sucrose preference test was evaluated in the mice with 50 min of MCAO (**C**) and the mice with 60 min of MCAO (**F**). *P < 0.05. N = 11–12.

In order to determine the optimal ischemic time for the PSD animal model, we further performed weekly depression-like behavioral tests, up to 4 weeks, after spatial restraint stress. Because mice with MCAO began to eat food regularly after 3 days of recovery, the ischemia-reperfusion + stress group (SIR). group was spatially restrained for 2 h daily for 2 to 4 weeks from the fourth day after MCAO. For the mice that underwent 50 min of MCAO, significant differences between the ischemia-reperfusion group (IR), and SIR groups were observed at the fourth week for tail suspension test (TST), the third week for forced swimming test (FST), and the fourth week for sucrose preference test (P < 0.05, Fig. 2A–C). However, for the mice that underwent 60 min of MCAO, spatial restraint stress for 1–2 weeks resulted in significant differences in performance on all the three tests between the IR group and SIR group (P < 0.05, Fig. 2D–F). Compared with stress-alone group, the mice in the SIR group had more severe depressive behaviors in the second week as shown in forced swimming test and sucrose preference test (P < 0.05, Fig. 2E,F). The results indicated that 60 min of MCAO was more effective in inducing PSD, and therefore 60 min of MCAO was chosen to establish the PSD mouse model and used in the following experiments.

### PSD exacerbated neurological function deficits

**Figure 3:**
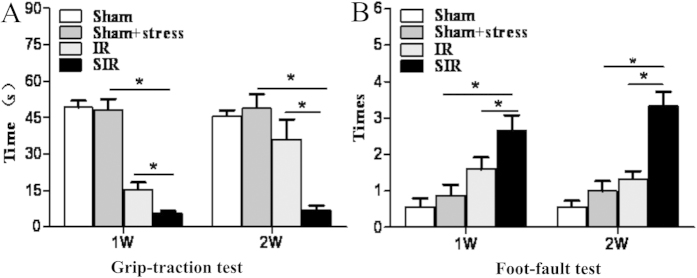
Spatial restraint stress exacerbated neurological functional deficits. After 60 min of MACO and spatial restraint stress, neurological function was examined. The difference in performance in the foot-fault test between both sides were shown (**A**). In the modified grip-traction test, the latency to fall from the rope was recorded (**B**). N = 11–12. *P < 0.05.

Previous studies have shown that depression exacerbates infarction size in patients and is a risk factor for stroke^18^. Furthermore, it is also known that treatment with anti-depressants promotes neurological functional recovery^19^. Therefore, we examined if untreated PSD delayed neurological function recovery in this model. The foot-fault test and the modified grip-traction test are widely used to evaluate neurological sensorimotor deficits and the muscle strength change after brain injury^20^. We found that the number of foot-faults was significantly increased in the SIR group (P < 0.05, Fig. 3A). Similarly, the hanging time in the SIR group was significant shorter than that in the IR group (P < 0.05, Fig. 3B).

### Decreased serotonin and dopamine levels in PSD

**Figure 4:**
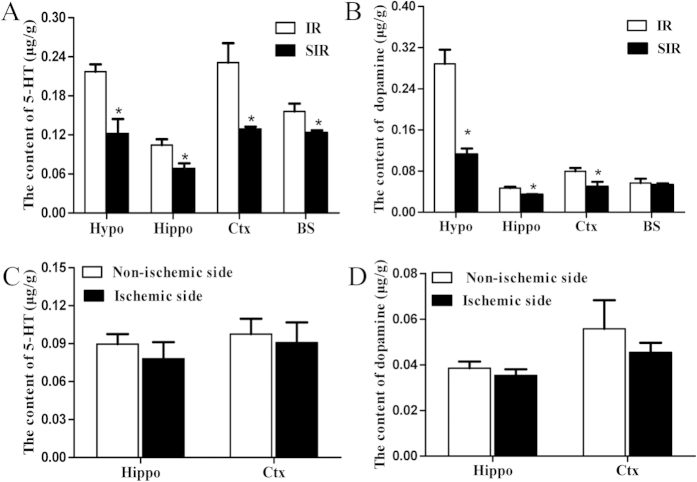
The neurotransmitters serotonin and dopamine decreased in different brain regions in stress-induced mice. Levels of serotonin were examined in the cortex (Ctx), the hippocampus (Hippo), the hypothalamus (Hypo), and the brainstem (BS) in the IR and SIR groups by HPLC (**A**). The levels of dopamine were also examined in the cortex, the hippocampus, the hypothalamus, and the brainstem regions in the IR and SIR groups (**B**). The levels of serotonin (**C**) and dopamine (**D**) were also examined in the cortex and hippocampus between ischemic and non-ischemic side. *P < 0.05 versus IR group. N = 4–6.

It has been reported that neurotransmitters such as dopamine and serotonin are decreased in human patients with depression^21^ and in pre-clinical animals with depressive behaviors^22^. To verify the decrease of depression-related neurotransmitters, we examined the levels of serotonin in different brain regions including the cortex, the hippocampus, the hypothalamus, and the brainstem. HPLC results indicated that serotonin levels were significantly decreased in all brain regions in SIR group mice (P < 0.05, Fig. 4A). Similarly, dopamine levels were also significantly decreased in the SIR group except in the brainstem region (P < 0.05, Fig. 4B). Interestingly, there was no significant difference in the levels of dopamine and 5-HT in the cortex and the hippocampus between the ischemic side and the non-ischemic side (p > 0.05, Fig. 4C,D).

### Regional brain BDNF levels was decreased in PSD mice

**Figure 5:**
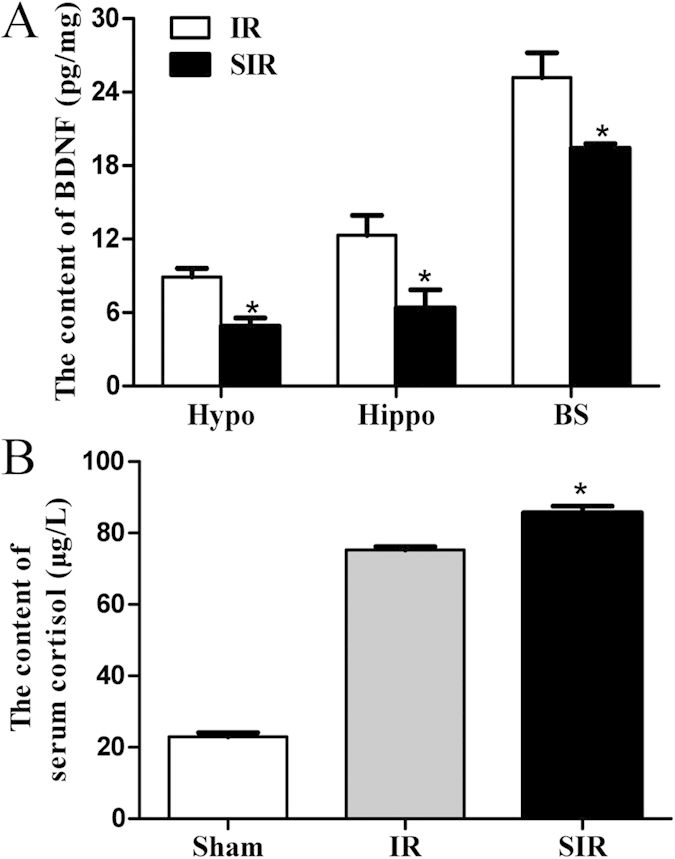
Decreased BDNF and increased serum cortisol were found in SIR mice. The BDNF level in brain regions including the hippocampus, the hypothalamus, and the brainstem was determined (**A**). The concentration of serum cortisol was also determined in the Sham, IR and SIR groups by ELISA (**B**). N = 3. *P < 0.05 versus Sham or IR group.

Recent studies suggest that depressed patients have decreased levels of BDNF in serum^23^ and BDNF is implicated in neurogenesis in the hippocampus^24^. To validate the change of BDNF in this model of PSD, we determined the levels of BDNF in different brain regions including the hippocampus, the hypothalamus, and the brainstem using an ELISA kit. As shown in Fig. 5A, BDNF levels decreased in all brain regions examined (P < 0.05).

### Serum cortisol levels were increased in PSD mice

Because disturbances of the hypothalamic–pituitary–adrenal axis (HPA) are one of the most consistent symptoms in patients with major depression^25^, we hypothesized that the same neuroendocrine disturbances existed in this model of PSD. To test this hypothesis, we directly measured serum cortisol levels in the control mice and the mice with PSD. Our results indicated that levels of serum cortisol in the SIR group were significantly increased (P < 0.05, Fig. 5B), indicating neuroendocrine disturbances in these mice.

### Body weight was decreased in mice with PSD

**Figure 6:**
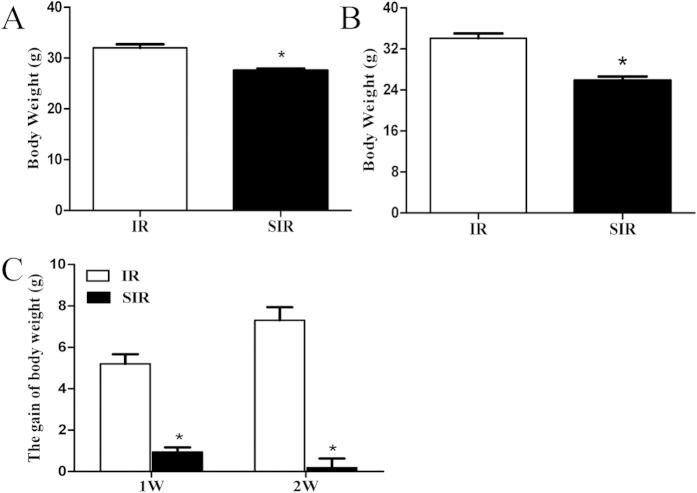
Mice in SIR group had lower body weight. The body weights were measured and recorded at the first week (**A**) and the second week (**B**) after spatial restraint stress in the IR group and the SIR group. The gain of body weight in the IR and SIR groups was analyzed (**C**). *P < 0.05, versus IR group. N = 8–11.

Body weight change may be a proxy measure of appetite change, which is a diagnostic criterion of depression^26^. Mice in the IR and SIR groups were weighed weekly after completion of spatial restraint stress. As shown in the first week (Fig. 6A) and the second week (Fig. 6B), compared with the IR group, body weight in the SIR group was significantly decreased (P < 0.05). At the same time, the gain of body weight was also reduced in the SIR group compared with the IR group (P < 0.05, Fig. 6C).

### Imipramine improved depressive behaviors in PSD mice

**Figure 7:**
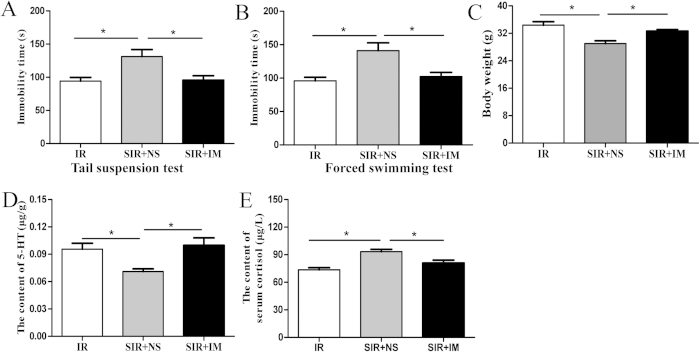
Imipramine rescued depressive behaviors and reversed biochemical changes. After mice in the SIR group were treated with imipramine (20 mg/kg) for 14 days, the immobility time in tail suspension test (**A**) and forced swimming test (**B**) were determined. The body weight in different group mice was recorded (**C**). The content of 5-HT was detected by HPLC (**D**). The concentration of serum cortisol was determined by ELISA (**E**). *P < 0.05 versus SIR + NS group. N = 4–6.

We examined whether the depressive behaviors of the mice in the SIR group could be improved by the anti-depressant drug imipramine. After 2 weeks of spatial restraint stress, mice in the SIR group were treated with imipramine (20 mg/kg) or saline for 14 days. Behavioral tests were performed post-treatment. Immobility time was significantly reduced in forced swimming test and tail suspension test in imipramine-treated SIR mice (P < 0.05, Fig. 7A,B). Compared with saline-treated mice in SIR group, the body weight of imipramine-treated mice markedly increased (P < 0.05, Fig. 7C). In order to verify whether imipramine increases the level of neurotransmitters, we detected 5-HT level by HPLC. Our results indicated that imipramine significantly increased 5-HT level in the hippocampus compared with saline-treated SIR mice (P < 0.05, Fig. 7D). In addition, serum cortisol level was also significantly decreased in the imipramine-treated SIR mice (P < 0.05, Fig. 7E).

## Discussion

The mechanism of PSD is complex and remains incompletely understood. The combined application of MCAO and unpredictable CMS has been demonstrated to cause depression-like behaviors in rodents and used to study the mechanisms of PSD^27^,^28^. However, the procedures of CMS are complex and composed of a variety of different conditions such as forced swimming, food/water deprivation, and cage tilting. While CMS causes depressive behaviors, the applied stressors such as electric shock and cold water could also lead to neurological damage^29^. In addition, as hemiplegia is the most common symptom following ischemic stroke and causes movement disturbances and psychological stress, a model which mimicks these conditions would demonstrate improved fidelity to real-world situations.

In our study, we applied MCAO and a repeated spatial restraint stress to demonstrate for the first time the establishment of a novel animal model of PSD, which more accurately reflects post-stroke symptoms. We also determined that 60 min is the optimal ischemic time period for inducing depressive behaviors, (Figs 1 and 2), as 70 min of MCAO caused high mortality, and 50 min was relatively less effective at inducing symptoms.

The criteria of ideal animal models should feature strong phenomenological similarities and similar pathophysiology, comparable etiology, and be responsive to common treatment^30^. Our study showed that spatial restraint stress following MCAO, mimicking real-world stroke-induced hemiplegia symptoms, caused depressive symptoms in mice, which is in agreement with previous study that chronic restraint stress is an important risk factor for the development of neuropsychiatric disorders^31^. This technique was simple and easily feasible, and induced depressive behaviors in mice in a short time period (1–2 weeks).

Forced swimming test and tail suspension test are widely used to validate animal models of depression and antidepressant-like effects in rodents^22^,^32^,^33^,^34^,^35^, although with concerns regarding the validity of these tests^36^. Mice with PSD exhibited severe neurological deficits including reduced muscle strength and decreased motor coordination (Fig. 3). Anhedonia is a core symptom of depression and is often assessed by sucrose preference. Body weight is a reflection of appetite, and was found to decrease. In this study, all the depressive-like behaviors were observed in the animal model after 1–2 weeks of spatial restraint stress.

Consistent with changes in patients with major depressive disorder, we found that the mice with PSD had reduced levels of BDNF and monoamine neurotransmitters such as serotonin and dopamine in different brain regions. Monoamine neurotransmitters in the brain, serotonin (5-HT) and dopamine (DA), are biogenic amines to transmit important information between nerve cells and effector cells and integrate the overall coordination of bodily functions. In addition, by affecting the normal function of the nervous system, changes in neurotransmitters can result in depressive symptoms^37^. In the present study, both the neurotransmitters decreased in the mice with PSD. In addition, elevated serum cortisol was also found after 2 weeks of spatial restraint stress (Figs 4, 5, 6), indicating the involvement of the HPA axis, which has been previously demonstrated to be induced by depressive symptoms^25^. Finally, these mice also responded to a known antidepressant, imipramine, which decreased immobility time and increased body weight (Fig. 7).

Since it is very difficult to validate depression in animal models, an appropriate animal model of post-stroke depression should fulfill as many criteria or endophenotypes as possible, such as anhedonia, behavioral despair, neuroendocrine disturbances, and changes in body weight. Our findings demonstrated that the combination of MCAO and spatial restraint stress result in a novel and feasible experimental model for PSD.

In summary, the combination of 60 minutes of MCAO and spatial restraint stress caused depressive behaviors in mice. This model had a high animal survival rate and fulfilled multiple criteria of major depressive disorder. Due to its restricted movement parameter, this model represents an ideal model for studying the mechanisms of PSD and future therapies of PSD.

## Methods

### Animals

Male ICR mice (28–30 g) were purchased from SLAC Company (Shanghai, China). The mice were maintained on 12 hour light/dark cycle. All animal procedures involving the use of animals for this study were approved by the University Committee on Animal Care of Soochow University and conducted in accordance with the guidelines of Animal Use and Care of the National Institutes of Health.

### The MCAO mouse model

Cerebral ischemic injury was induced by the model of middle cerebral artery occlusion as described by Longa *et al*.^38^. Briefly, the mice were anesthetized by 4% chloral hydrate (0.01 ml/g body weight) via intraperitoneal (i.p.) injection. The right common carotid artery (CCA), the right external carotid artery (ECA) and the internal carotid artery (ICA) were exposed through a ventral midline neck incision. After the CCA was clamped and the ECA was ligatured by silk sutures, the ECA was cut 2 mm distal to the ECA–CCA branch. A 6–0 nylon monofilament (Ethilon, Ethicon Inc) coated with silicon resin (Heraeus, Kulzer, Germany) was inserted intraluminally into the right CCA 9–11 mm distal to the origin of middle cerebral artery until a faint resistance was detected. Reperfusion was achieved by withdrawing the suture after MCAO at indicated time (50, 60 or 70 min) to restore blood supply of the MCA territory. Body temperature was maintained at 36.5–37.5 °C using a heating pad on the surgical table throughout the procedure from the start of the surgery until the animals revived from anesthesia. To monitor ischemia and reperfusion, the local cerebral blood flow was measured using a Laser-Doppler blood flowmeter (Periflux 5010, PERIMED, Sweden) positioned at 1 mm posterior and 3 mm lateral to the Bregma.

### Experimental groups

Following 3 days of MCAO, animals were randomly assigned to different experimental groups: sham group (Sham), stress-alone group (Stress); ischemia-reperfusion group (IR), ischemia-reperfusion + stress group (SIR). Mice in the IR group underwent MCAO with no subsequent spatial restraint stress. Mice in Stress group and SIR group were subject to spatial restraint stress in restrainers between 9 am and 11 am daily for 2 weeks from the fourth days after MCAO.

### Neurological deficit scoring evaluation

Neurological deficits were evaluated at 24 hours after the MCAO according to a graded scoring system described previously^39^: specifically, a score of 0 equates to no deficit; a score of 1 equates to flexion of the contralateral torso and forelimbs; a score of 2 equates to turning to the ipsilateral side when held by tails; a score of 3 equates to leaning toward the affected side; a score of 4 equates to no spontaneous locomotor activity. If no deficit was observed after MCAO, the animal was removed from further study.

### Spatial restraint stress

Mice were individually placed into a modified well-ventilated 50-ml centrifuge tube daily from 9 am to 11 am for 2 h per day and were not able to move forward or backward in tubes. Mice in Sham group and IR group remained undisturbed in their original cages. After restraint stress, mice were removed from the tube and returned to their original cages.

### Modified grip-traction test

Muscle strength was tested by a modified grip-traction test as previously described^20^. The ability to hang on to a horizontal rope (0.6-cm diameter plastic tube placed horizontally at 45 cm above the table) by the forepaws was evaluated in terms of time to fall, up to a maximum of 60 seconds.

### Foot-fault Test

We used an elevated (1 m) grid floor (50 × 40 cm) with a wire diameter of 0.4 cm to examine the number of foot-faults according to previously described methods^20^. A foot-fault was defined as a slip of the paw off the grid bars as a result of a misplaced limb during movement. The difference of left (contralateral foot-faults) to right (ipsilateral foot-faults) was also calculated.

### Forced Swimming Test (FST)

Forced swimming test was performed as described previously^40^. Each mouse was placed in a glass cylinder (20 cm high, 15 cm in diameter) with water (23–25 °C, 14 cm in depth) for 6 min. Immobility time was recorded when mouse floated or made minimum movement necessary to maintain floating in the water.

### Tail Suspension Test (TST)

The tail suspension test was performed according to the method outlined in previous reports^41^ with minor modifications of elevating the mouse 45 cm above the desktop. Each mouse was suspended for 6 min and the immobility time was recorded.

### Sucrose Preference Test

The sucrose preference test was performed as described previously^22^. Briefly, mice were housed individually and trained to drink water from two bottles for 24 h. The next day, a bottle of water was replaced with a bottle of 1% sucrose solution. After 24 hours, the positions of two bottles were exchanged. On the fourth day, mice were deprived of water and food for 24 hours. Before water and food were supplied, the bottles were weighted. After 24 hours, the bottles were weighed again to calculate fluid consumption. The percentage of consumed sucrose to total drink was also calculated.

### Body Weight

Body weight was measured by electronic balance and recorded at the same time point every 7 days. On the 7th day, all mice were weighed before undergoing spatial restraint stress.

### High Performance Liquid Chromatography (HPLC)

Serotonin and dopamine levels in brain regions were detected as described previously^22^. The hypothalamus and the brainstem were easily separated from the mouse brain. For the hippocampus and the cortex, these tissues were separated as described previously^42^,^43^. Brain regions were dissected on ice. Tissues were homogenized after addition of 200 ml perchloric acid (0.4 M). The homogenates were placed on ice and then centrifuged at 10,000 g for 15 min. Perchloric acid (0.4 M) was added to 1 ml and injected into an HPLC system. Six mice in each group were used for the analysis of monoamine levels.

### Measurement of Brain Derived Neurotrophic Factor (BDNF) and Cortisol

BDNF levels in brain tissues were determined by an enzyme-linked immunosorbent assay kit (Merck Systems; Germany). Brain tissues were homogenized and collected for the determination of BDNF according to the assay kit instructions. Serum cortisol was also determined by an enzyme-linked immunosorbent assay kit (China). Absorbance was measured at 450 nm using a microplate absorbance reader (Tecan infinite M200 PRO, Austria).

### Administration of Imipramine

Imipramine is a well-known tricyclic antidepressant that is commonly used to treat depression^44^,^45^. Before every usage, imipramine (20 mg/kg, Sigma-Aldrich Corp., St. Louis, USA) was dissolved in normal saline. After 2 weeks of restraint stress, mice in the SIR group received (i.p.) imipramine at a 20 mg/kg dose or normal saline.

### Statistical Analysis

All data were expressed as mean ± SEM. Differences between two groups were determined with Student’s T test. The differences among groups were compared with one-way analysis of variance followed by Turkey’s multiple-comparison test. P < 0.05 was considered statistically significant.

## Additional Information

**How to cite this article**: Zhang, G. *et al*. Combined use of spatial restraint stress and middle cerebral artery occlusion is a novel model of post-stroke depression in mice. *Sci. Rep.* **5**, 16751; doi: 10.1038/srep16751 (2015).
